# Effects of Lipooligosaccharide Inner Core Truncation on Bile Resistance and Chick Colonization by *Campylobacter jejuni*

**DOI:** 10.1371/journal.pone.0056900

**Published:** 2013-02-20

**Authors:** Taketoshi Iwata, Kazuhiro Chiku, Ken-ichi Amano, Masahiro Kusumoto, Mayumi Ohnishi-Kameyama, Hiroshi Ono, Masato Akiba

**Affiliations:** 1 Bacterial and Parasitic Disease Research Division, National Institute of Animal Health, Tsukuba, Ibaraki, Japan; 2 Analytical Science Division, National Food Research Institute, Tsukuba, Ibaraki, Japan; 3 Bioscience Research Education Center, Akita University, Akita, Akita, Japan; 4 Graduate School of Life and Environmental Sciences, Osaka Prefecture University, Izumisano, Osaka, Japan; Charité-University Medicine Berlin, Germany

## Abstract

*Campylobacter jejuni* is the most common bacterium that causes diarrhea worldwide, and chickens are considered the main reservoir of this pathogen. This study investigated the effects of serial truncation of lipooligosaccharide (LOS), a major component of the outer membrane of *C. jejuni*, on its bile resistance and intestinal colonization ability in chickens. Genes encoding *manno*-heptose synthetases or glycosyltransferases were inactivated to generate isogenic mutants. Serial truncation of the LOS core oligosaccharide caused a stepwise increase in susceptibilities of two *C. jejuni* strains, NCTC 11168 and 81-176, to bile acids. Inactivation of *hldE, hldD*, or *waaC* caused severe truncation of the core oligosaccharide, which greatly increased the susceptibility to bile acids. Both wild-type strains grew normally in chicken intestinal extracts, whereas the mutants with severe oligosaccharide truncation were not detected 12 h after inoculation. These mutants attained viable bacterial counts in the bile acid-free extracts 24 h after inoculation. The wild-type strain 11-164 was present in the cecal contents at >10^7^ CFU/g on 5 days after challenge infection and after this time period, whereas its *hldD* mutant was present at <10^3^ CFU/g throughout the experimental period. *Trans*-complementation of the *hldD* mutant with the wild-type *hldD* allele completely restored the *in vivo* colonization level to that of the wild-type strain. Mutants with a shorter LOS had higher hydrophobicities. Thus, the length of the LOS core oligosaccharide affected the surface hydrophobicity and bile resistance of *C. jejuni* as well as its ability to colonize chicken intestines.

## Introduction

*Campylobacter* are curved-to-spiral shaped, flagellated gram-negative rods that grow under microaerophilic or anaerobic conditions [1]. Of the 25 species in the genus [2], *C. jejuni* and its close relative *C. coli* are the most important foodborne pathogens (hereafter collectively referred to as *Campylobacter*). *Campylobacter* is one of the most common bacterial causes of diarrhea in industrialized and developing countries, with approximately 400 million cases per year worldwide [3]. The annual economic burden of *Campylobacter* infections, based on medical costs and productivity loss in the USA, was estimated to be 1.5–8.0 billion dollars [4]. Campylobacteriosis is a typical self-limited enteritis, although *C. jejuni* strains with specific lipooligosaccharide (LOS) structures are known causative agents of an acute neuromuscular paralysis, Guillain–Barré syndrome, which develops 1–2 weeks after infections [5]. Although *Campylobacter* bacteremia is uncommon, systemic *C. jejuni* infections have been reported in the elderly, infants younger than 12 months, and patients with underlying conditions such as liver cirrhosis, human immunodeficiency virus disease, and therapy-induced immunosuppression [6], [7].

*C. jejuni* colonizes the intestinal tracts of various wild and domestic animals, and it persists in untreated and adequately treated aquatic environments. Avian species such as poultry and wild birds are considered the main reservoir of *C. jejuni* [8]–[11]. Most cases of campylobacteriosis are associated with the consumption of contaminated raw or undercooked poultry meat or other foods contaminated by these items through preparations [12]. An ice water immersion chilling step is suspected to be a major cause of fecal contamination during chicken meat processing [13]. The reduction of *C. jejuni* contamination in the food chain is an important step in the control of campylobacteriosis. One approach is to prevent *C. jejuni* colonization of broiler chickens. To successfully colonize chicken intestinal tracts, *C. jejuni* needs to tolerate various environmental stresses such as pH variation, low oxygen, nutrient limitation, elevated osmotic pressure, and digestive fluids including bile acids [14]. Understanding these stress resistance mechanisms may help to develop novel measures to control *Campylobacter* colonization in chickens.

In humans, bile is produced in the liver and stored in the gall bladder. After the ingestion of food, bile is secreted from the gall bladder into the duodenum, which helps digestion and absorption of dietary fats and fat-soluble vitamins. Bile consists of bile acids, pigments, phospholipids, and cholesterol. Bile acids are synthesized from cholesterol via a multienzyme process and promote fat absorption by producing polymolecular aggregates known as micelles [15]. Bile acids display antimicrobial activity by inducing membrane damage and oxidative stress to bacterial DNA [16], [17]. To overcome the antimicrobial effect of bile acids, enteric bacteria have evolved multiple mechanisms including active efflux, modulation of the synthesis of lipopolysaccharide (LPS) and porins, and production of bile acid hydrolase [16], [17]. Active efflux by the CmeABC system is a well-characterized bile resistance mechanism in *C. jejuni*. Functional disruption of this system results in substantial decreases in *C. jejuni* resistance to various antimicrobials including bile acids [18] and the loss of its ability to colonize chicken intestinal tracts [19]. The relationship between outer membrane integrity and bile resistance of *C. jejuni* remains unclear.

LOS is a major component of the outer membrane of gram-negative bacteria, including the genera *Neisseria*, *Haemophilus*, *Bordetella*, *Branhamella*, and *Campylobacter* [20]. Unlike LPS, LOS lacks the repeating polysaccharide O antigen and is composed of covalently linked domains, i.e., lipid A, a hydrophobic anchor, and a core oligosaccharide (OS) that consists of inner and outer core regions. More specifically, two 3-deoxy-d-*manno*-octulosonic acid (Kdo) residues are linked to lipid A and two l-*glycero*-d-*manno*-heptose residues are linked to Kdo in the inner core region of LOS in *C. jejuni* strains for which the LOS structure has been determined previously. Kdo is highly conserved in gram-negative bacteria and is essential for cell growth [21], whereas l-*glycero*-d-*manno*-heptose is not [22]. Malfunctioning of ADP-l-*glycero*-d-*manno*-heptose synthetases or heptosyltransferases that truncate the inner core region of *C. jejuni* reduces virulence and increases the susceptibility of the microbe to several types of detergents [22]–[24]. Jeon et al. reported that mutation in *waaF*, a gene encoding a heptosyltransferase, in *C. jejuni* reduced minimum inhibitory concentration (MIC) of polymyxin B and sodium dodecyl sulfate (SDS), whereas the mutant showed no changes in MIC of choleate [23]. Naito et al. showed that a *waaF* mutant exhibited significantly reduced intestinal colonization in mice [25]. However, the effect of structural changes in LOS on the *C. jejuni* susceptibility to bile acids remains unclear.

In this study, we constructed *C. jejuni* mutants with serial LOS OS truncations and compared their susceptibilities to bile acids with those of the wild-type strains. Colonization of the chicken intestinal tract by the mutants was also compared with that by the wild-type strains. This work provides a greater understanding of the minimum LOS core structures in *C. jejuni* that facilitate the tolerance of this pathogen to bile acids and promote its colonization of chicken intestinal tracts.

## Results

### LOS mobilities of *C. jejuni* strains determined by tricine-SDS-PAGE

**Figure 1:**
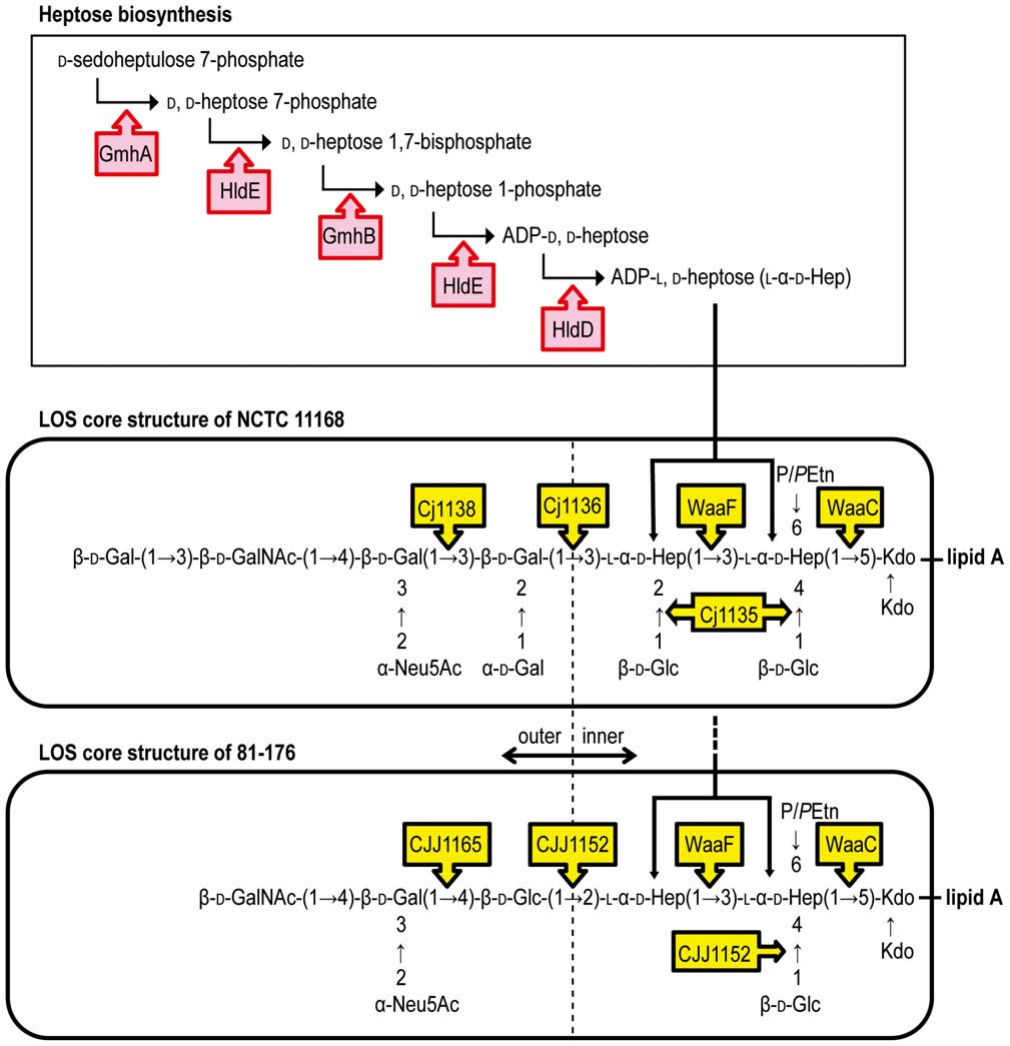
Hypothetical pathways of LOS biosynthesis in *C. jejuni* NCTC 11168 and 81-176. The enzymes are indicated by arrow boxes. Abbreviations: GalNAc, *N*-acetylgalactosamine; Neu5Ac, *N*-acetylneuraminic acid; Gal, galactose; Glc, glucose; Hep, heptose; P/*P*Etn, phosphate or pyrophosphoethanolamine; Kdo, 2-keto-3-deoxy-d-*manno*-octulosonic acid.

Isogenic mutants of the following genes were constructed: *gmhA* (*cj1149*), *hldE* (*cj1150*), *hldD* (*cj1151*), *waaC* (*cj1133*), *waaF* (*cj1148*), *cj1135*, *cj1136*, and *cj1138* in *C. jejuni* NCTC 11168; and *gmhA*, *hldE*, *hldD*, *waaC*, *waaF*, *cjj1152*, and *cjj1165* in *C. jejuni* 81-176 (Table 1). These genes were predicted to code for *manno*-heptose synthetases or glycosyltransferases, as indicated in Fig. 1 [25]–[27]. *gmhB* (*cj1152*) is essential for *C. jejuni* [22] and therefore was not inactivated in either of the strains used in this study. Complementations were performed for all LOS-truncated mutants.

**Table 1:** Plasmids and bacterial strains used in this study.

Plasmid or strain	Description	Source or reference
Plasmids		
pGem-T Easy	PCR cloning vector, Amp^r^	Promega
pUOA18	*E. coli*-*C. jejuni* shuttle vector, Cm^r^	[38]
pUOA18Km	pUOA18 modified to replace *cat* with *kan*	This study
pUOA18Km-*hldE*	pUOA18Km with *hldE* (*cj1150*)	This study
pUOA18Km-*hldD*	pUOA18Km with *hldD* (*cj1151*)	This study
pUOA18Km-*waaC*	pUOA18Km with *waaC* (*cj1133*)	This study
pUOA18Km-*waaF*	pUOA18Km with *waaF* (*cj1148*)	This study
pUOA18Km-*cj1135*	pUOA18Km with *cj1135*	This study
pUOA18Km-*cj1136*	pUOA18Km with *cj1136*	This study
pUOA18Km-*cj1138*	pUOA18Km with *cj1138*	This study
pUOA18Km-*cjj1152*	pUOA18Km with *cjj1152*	This study
pUOA18Km-*cjj1165*	pUOA18Km with *cjj1165*	This study
pRK2013	helper plasmid for triparental mating, Km^r^	[41]
Strains		
* C. jejuni* parental strains		
NCTC 11168	Wild type; human isolate	[28]
81-176	Wild type; human isolate	[55]
11-164	Wild type; chicken isolate, Nal^r^	This study
*C. jejuni* mutant strains		
168gmhA	NCTC 11168 derivative; *gmhA* mutant	This study
168hldE	NCTC 11168 derivative; *hldE* mutant	This study
168hldD	NCTC 11168 derivative; *hldD* mutant	This study
168waaC	NCTC 11168 derivative; *waaC* mutant	This study
168waaF	NCTC 11168 derivative; *waaF* mutant	This study
168cj1135	NCTC 11168 derivative; *cj1135* mutant	This study
168cj1136	NCTC 11168 derivative; *cj1136* mutant	This study
168cj1138	NCTC 11168 derivative; *cj1138* mutant	This study
168kpsS	NCTC 11168 derivative; *kpsS* mutant	This study
168hldEc	168hldE/pUOA18Km-*hldE*	This study
168hldDc	168hldD/pUOA18Km-*hldD*	This study
168waaCc	168waaC/pUOA18Km-*waaC*	This study
168waaFc	168waaF/pUOA18Km-*waaF*	This study
168cj1135c	168cj1135/pUOA18Km-*cj1135*	This study
168cj1136c	168cj1136/pUOA18Km-*cj1136*	This study
168cj1138c	168cj1138/pUOA18Km-*cj1138*	This study
817gmhA	81-176 derivative; *gmhA* mutant	This study
817hldE	81-176 derivative; *hldE* mutant	This study
817hldD	81-176 derivative; *hldD* mutant	This study
817waaC	81-176 derivative; *waaC* mutant	This study
817waaF	81-176 derivative; *waaF* mutant	This study
817cjj1152	81-176 derivative; *cjj1152* mutant	This study
817cjj1165	81-176 derivative; *cjj1165* mutant	This study
817kpsS	NCTC 11168 derivative; *kpsS* mutant	This study
817hldEc	817hldE/pUOA18Km-*hldE*	This study
817hldDc	817hldD/pUOA18Km-*hldD*	This study
817waaCc	817waaC/pUOA18Km-*waaC*	This study
817waaFc	817waaF/pUOA18Km-*waaF*	This study
817cjj1152c	817cjj1152/pUOA18Km-*cjj1152*	This study
817cjj1165c	817cjj1165/pUOA18Km-*cjj1165*	This study
164hldE	11-164 derivative; *hldE* mutant	This study
164hldD	11-164 derivative; *hldD* mutant	This study
164waaC	11-164 derivative; *waaC* mutant	This study
164waaF	11-164 derivative; *waaF* mutant	This study
164hldDc	164hldD/pUOA18Km-*hldD*	This study
*E. coli* DH5α	cloning strain	Takara

**Figure 2:**
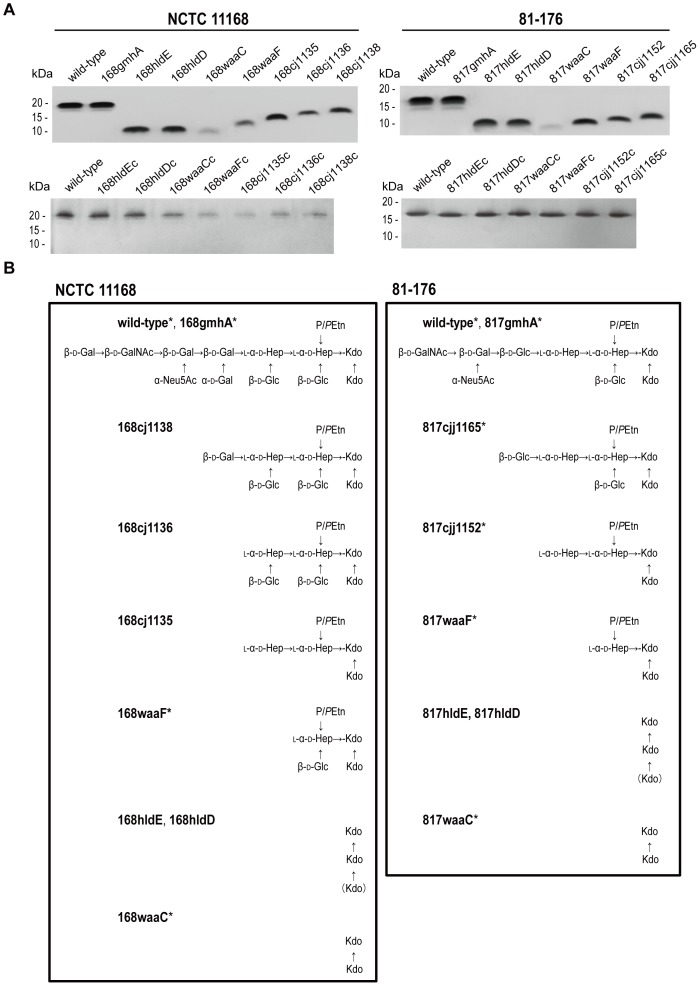
LOS profiles and LOS OS structures of the *C. jejuni* wild-type strains (NCTC 11168 and 81-176) and mutants. (A) LOS samples were analyzed by tricine-SDS-PAGE using 16% tricine gels and visualized by silver staining. The positions of the protein size markers are indicated on the left of each panel. (B) The LOS structures of 168hldE, 168hldD, 168cj1135, 168cj1136, 168cj1138, 817hldE, and 817hldD were analyzed by MALDI-TOF MS and GC-MS in this study. The wild-type strains and other mutants with asterisks have structures similar to those determined in previous studies [24]–[26], [52]–[54]. Abbreviations, refer to the legend of Fig. 1.

To confirm LOS truncations in each mutant, LOS samples were analyzed by tricine-SDS-PAGE, followed by silver staining (Fig. 2A). LOS produced by each mutant constructed in this study migrated more quickly than that produced by wild-type strains, with the exceptions of 168gmhA and 817gmhA. A previous whole genome analysis showed that NCTC 11168 contained two copies of the heptose isomerase gene: *gmhA* (*cj1149* located in the LOS gene cluster) and *gmhA2* (*cj1424* located in the Capsular polysaccharide (CPS) gene cluster) [28]. Karlyshev et al. [26] reported that mutations in either gene in NCTC 11168 did not affect LOS and CPS expression, whereas mutation in both *gmhA* and *gmhA2* resulted in the loss of CPS 6-O-methylheptose and LOS truncation.

Glycosyltransferase mutants, i.e., 168waaC, 168waaF, 168cj1135, 168cj1136, 168cj1138, 817waaC, 817waaF, 817cjj1152, and 817cjj1165, exhibited increased LOS mobility in a stepwise manner, as reported previously [25], [26]. The mobility of LOS produced by the *hldE* and *hldD* mutants was identical and was slightly lower than that of LOS produced by the *waaC* mutant derived from NCTC 11168 and 81-176. The LOS bands of 168waaC and 817waaC were faint compared with those of other strains. Thus, the surface properties of these strains may be different from those of other strains. The LOS migration characteristics of all complemented strains were identical to those of their wild-type strains.

### LOS structures of *C. jejuni* mutants determined by mass spectrometry

The LOS structures of 168hldE, 168hldD, 168cj1135, 168cj1136, 168cj1138, 817hldE, and 817hldD were determined by matrix-assisted laser desorption/ionization time-of-flight mass spectrometry (MALDI-TOF MS) and gas chromatography-mass spectrometry (GC-MS) for the first time in the present study and are shown in Fig. 2B. OS produced by 168cj1138 had an intact inner core with one galactose residue, whereas OS produced by 168cj1136 lacked all residues on the outer core. 168cj1135 contained no glucose or galactose residues in its OS, and the truncated molecule comprised heptose, Kdo, and phosphate or pyrophosphorylethanolamine (P/*P*Etn) (Table S1). The MALDI-TOF MS spectra of the intact LOS produced by the *hldE* (168hldE and 817hldE) and *hldD* (168hldD and 817hldD) mutants contained four intense ions, with one ion corresponding to the lipid A fragment ([M-H-H_2_O] at *m/z* 1904), and the intervals were observed at Δ*m*/*z* 219–222 (Table S2). Only Kdo was detected in the GC mass spectra, whereas l-*glycero*-d-*manno*-heptose and d-*glycero*-d-*manno*-heptose were not observed in the OS samples obtained from the mutants.

### Effects of LOS truncations on antimicrobial resistance

As shown in Table 2, MICs of cholic acid, deoxycholic acid, and taurocholic acid for 168waaF, 168cj1135, 168cj1136, and 168cj1138 were ≥2-fold smaller than those for NCTC 11168 and 168gmhA, while no difference was observed for other bile acids. MICs of the five bile acids used in this study for 168hldE, 168hldD, and 168waaC were 4- to >32-fold lower than those for NCTC 11168 and 168gmhA. The results obtained for *C. jejuni* 81-176 and its mutants were similar to those obtained for NCTC 11168 and its mutants, although 817waaF showed a 2- to >16-fold increase in its susceptibility to all five bile acids. Some of the LOS-truncated mutants had increased susceptibilities to erythromycin (1- to 8-fold), rifampin (1- to 2-fold), polymyxin B (2-fold), or SDS (1- to 2-fold). No difference was observed in the susceptibility to the other antimicrobials tested. The susceptibilities of 168gmhA and 817gmhA, and all complemented strains to bile acids and other antimicrobials were the same as those of the wild-type strains.

**Table 2:** Antimicrobial susceptibility of *C. jejuni*.

Antimicrobial^a^	MIC (µg/ml)^b^							
	NCTC 11168				81-176			
	Ia	IIa	IIIa	IVa	Ib	IIb	IIIb	IVb
	(wild-type, 168gmhA)	(168cj1136, 168cj1138)	(168waaF, 168cj1135)	(168hldE, 168hldD, 168waaC)	(wild-type, 817gmhA)	(817cjj1152, 817cjj1165)	(817waaF)	(817hldE, 817hldD, 817waaC)
**Ox gall**	25,000	25,000	25,000	6,250	12,500	12,500	6,250	6,250
**Ox bile extract**	12,500	12,500	12,500	3,130	6,250	6,250	3,130	3,130
**Cholic acid**	6,250	3,130	3,130	1,560	3,130	3,130	780	780
**Deoxycholic acid**	>10,000	10,000	5,000	313	>10,000	10,000	625	313
**Taurocholic acid**	>100,000	100,000	100,000	12,500	>100,000	100,000	12,500	12,500
**SDS**	100	100	100	50	50	50	25	25
Ampicillin	>50	>50	>50	>50	3.13	3.13	3.13	3.13
Cefsulodin	25	25	25	25	12.5	12.5	12.5	12.5
Gentamicin	0.313	0.313	0.313	0.313	0.313	0.313	0.313	0.313
Tetracycline	<0.2	<0.2	<0.2	<0.2	6.25	6.25	6.25	6.25
**Erythromycin**	0.5	0.125	0.125	0.0625	0.125	0.125	0.0625	0.0313
Trimethoprim	500	500	500	500	250	250	250	250
Nalidixic acid	10	10	10	10	2.5	2.5	2.5	2.5
Enrofloxacin	0.0625	0.0625	0.0625	0.0625	0.0313	0.0313	0.0313	0.0313
**Rifampin**	200	200	200	100	100	100	50	50
**Polymyxin B**	5	2.5	2.5	2.5	2.5	1.25	1.25	1.25

a Bold-faced types indicate that the differences in MICs observed between NCTC 11168 or 81-176 wild-type strain and any mutant.

b The NCTC 11168 or 81-176 wild-type strain and mutants were divided into four groups (Ia–IVa or Ib–IVb) on the basis of MICs. The strains belonging to the same group showed identical MIC values for all of the antimicrobials tested.

The multi-drug resistance system, CmeABC, is known to play an important role in bile resistance in *C. jejuni* [19]. The nucleotide sequences of *cmeABC* and its promoter region in the LOS-truncated mutants, 168cj1136, 168waaF, and 168hldD, were identical to those of the wild-type strain NCTC 11168, which suggested that CmeABC was functional in these mutants. CPS was detected by Alcian blue staining in all LOS-truncated strains constructed in this study and the wild-type strains (Fig. S1). However, inactivation of *kpsS* (*cj1413*) encoding the CPS export protein did not affect bile resistance in both NCTC 11168 and 81-176; MICs of ox gall, ox bile extract, cholic acid, deoxycholic acid, and taurocholic acid were identical in the *kpsS* mutants and wild-type strains.

### Effects of LOS truncations on resistance to chicken intestinal extracts

**Figure 3:**
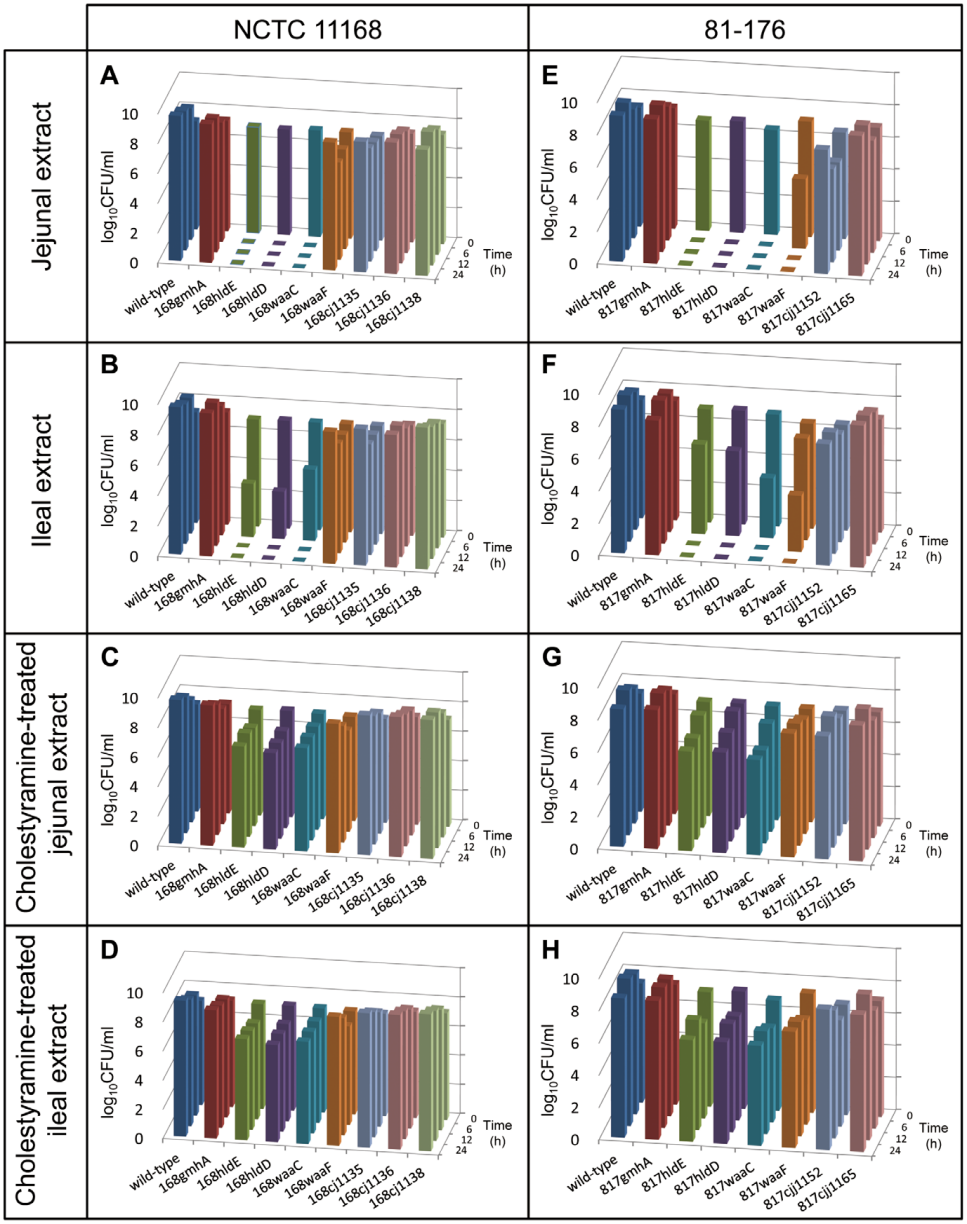
Growth of *C. jejuni* strains in chicken intestinal extracts. *C. jejuni* NCTC 11168 wild-type strain and its mutants were grown in chicken jejunal extract (A), chicken ileal extract (B), chicken jejunal extract pretreated with cholestyramine (C), and chicken ileal extract pretreated with cholestyramine (D). *C. jejuni* 81-176 wild-type strain and its mutants were also grown in chicken jejunal extract (E), chicken ileal extract (F), chicken jejunal extract pretreated with cholestyramine (G), and chicken ileal extract pretreated with cholestyramine (H). Each point represents the mean value obtained from triplicate wells in 96-well microtiter plates. The detection limit for the assay was 0.4 log_10_ CFU/ml.

The growth of the wild-type strains and mutants in intestinal extracts and cholestyramine-treated extracts were investigated to determine whether the bile acids in chicken intestinal extracts affected the viability of *Campylobacter*. Total bile acid concentrations in the jejunal and ileal extracts were 8.5 and 4.9 mM, respectively. After cholestyramine treatment, bile acid concentrations in the jejunal and ileal extracts were reduced to 1.8 mM, and 1.6 mM, respectively. The wild-type strain NCTC 11168 grew normally in both intestinal extracts, but the mutant strains, i.e., 168hldE, 168hldD, and 168waaC, were not detectable in the jejunal and ileal extracts 6 and 12 h after inoculation, respectively (Fig. 3A and 3B). In both intestinal extracts, the other mutants derived from NCTC 11168 were detectable 24 h after inoculation; however, the viable bacterial counts were lower than those of the wild-type strain 6 h after inoculation. In contrast, the 168hldE, 168hldD, and 168waaC mutants were detectable at all time points in the cholestyramine-treated extracts, and their viable bacterial counts were >1 log unit lower than those of the other strains 24 h after inoculation (Fig. 3C and 3D). Results obtained for the wild-type strain 81-176 and its mutants were similar to those obtained for NCTC 11168 and its mutants, except the 817waaF. This strain was not detectable 12 and 24 h after inoculation in the jejunal and ileal extracts, respectively (Fig. 3E and 3F), whereas 168waaF, the corresponding mutant derived from NCTC 11168, was detectable until 24 h after inoculation in both extracts.

### Effects of LOS truncations on chick cecum colonization

To evaluate the effects of LOS truncation on chick cecum colonization, we used strain 11-164 as the wild-type because colonization was better with this strain compared with strains NCTC 11168 and 81-176 in our preliminary experiments (data not shown). The *hldE*, *hldD*, *waaC*, and *waaF* mutants were constructed from strain 11-164 by natural transformation, and these mutants were used in the *in vitro* experiments including LOS analysis by mass spectrometry, LOS mobility, antimicrobial susceptibility, and surface hydrophobicity. All results were similar to those obtained with mutants of NCTC 11168 and 81-176. Inactivation of *hldE*, *hldD*, or *waaC* produced severe LOS truncations, which greatly increased the susceptibility of the strains to bile acids (Tables S2 and S3 and Fig. S2A).

Of these, 164hldD and the *hldD*-complemented strain, 164hldDc, were used in the chick colonization experiments. In 164hldD, the MALDI-TOF MS spectra of the intact LOS contained four intense ions, while only Kdo was detected in the GC mass spectra of the OS sample, as well as strains 168hldD and 817hldD (Table S2). No significant differences were observed among the strains in terms of their growth curves in Mueller–Hinton (MH) broth (Becton Dickinson and Company, Sparks, MD, USA) and the resistance to low pH stress (Fig. S3). In addition, the motilities of all LOS-truncated mutants of 11-164, including 164hldD, were identical to those of the wild-type strains in 0.4% soft MH agar (Becton Dickinson and Company) (Fig. S2B).

**Figure 4:**
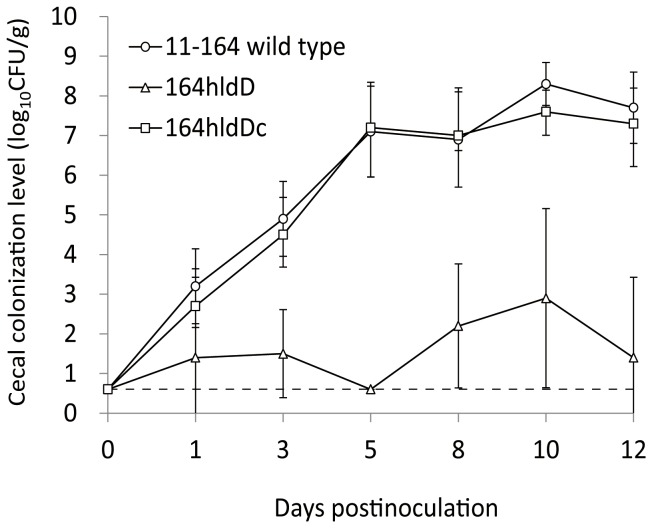
Colonization of chickens by the *C. jejuni* 11-164 wild-type strain and its isogenic *hldD* mutant. Each point represents the mean log_10_ CFU/g in the cecal contents of chickens in each group. Standard deviations are indicated by error bars. The dashed line indicates the lower limit of detection (0.7 log_10_ CFU/g).

As shown in Fig. 4, 11-164 and 164hldDc colonized chickens as early as 1 day after inoculation, and the viable bacterial counts were >10^7^ CFU/g on 5 days after inoculation and after this time period. The mutant strain 164hldD was also detected 1 day after inoculation, although the viable bacterial counts were <10^3^ CFU/g throughout the experimental period. 164hldD was not detected constantly in the cecal contents, whereas 11-164 and 164hldDc were detected in all samples recovered during this experiment.

### Effects of LOS truncations on cell surface hydrophobicity

It was reported that the surface hydrophobicity of the *waaF* mutant constructed from strain NCTC 11168 was significantly higher than that of the wild-type strain [23]. To test whether serial truncations of LOS OSs caused a stepwise elevation in the surface hydrophobicity, we conducted a bacterial adherence to hydrocarbon (octane) assay with the LOS-truncated mutants and their wild-type strains.

**Figure 5:**
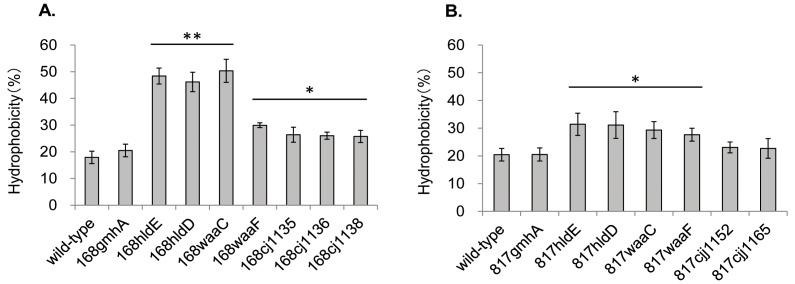
Effect of LOS truncations on the cell surface hydrophobicity of *C. jejuni* NCTC 11168 (A) and 81-176 (B). The percentage hydrophobicity of the bacterial surface was calculated as follows: (1−final OD_600_ of the aqueous phase/initial OD_600_ of the cell suspension)×100. Standard deviations are indicated by error bars. *Significantly different from wild-type, **significant differences from both the wild-type and LOS mutants are labeled with an asterisk (*P*<0.05).

As shown in Fig. 5A, the surface hydrophobicities of 168hldE, 168hldD, and 168waaC were 2.6- to 2.8-fold higher, while those of the other derivatives were 1.4- to 1.7-fold higher than the surface hydrophobicities of NCTC 11168. The surface hydrophobicities of 817hldE, 817hldD, 817waaC, and 817waaF were 1.4- to 1.5-fold higher than those of the wild-type strain, 81-176, and the difference was significant (Fig. 5B). The surface hydrophobicities of strains 168gmhA, 817gmhA, 817cjj1152, and 817cjj1165 were comparable to those of their wild-type strains (Fig. 5). Complementation restored hydrophobicity in all LOS-truncated mutants (data not shown).

Elevation of the surface hydrophobicity was also observed in mutants constructed from strain 11-164, which was used in the chicken colonization experiments; the surface hydrophobicity of 164hldE, 164hldD, 164waaC, and 164waaF was 1.6- to 1.9-fold higher than that of the wild-type strain (Fig. S2C).

## Discussion

The involvement of bacterial LPS in bile resistance has been reported [15], [16]. Deep rough mutants of *Escherichia coli* and *Vibrio cholerae* showed an increased susceptibility to bile acids [29], [30]. However, the relationship between the LPS/LOS OS structure and bile resistance in gram-negative bacteria remains largely unknown. Recently, Javed et al. [31] reported that the *cj1136* mutant of the hyperinvasive wild-type strain 01/51 had an increased susceptibility to sodium taurocholate and a reduced ability to colonize 1-day-old chicks. However, they did not test any other mutants with LOS OS truncations.

In this study, we constructed multiple *C. jejuni* mutants with serial LOS OS truncations and showed that differences in LOS OS length of one or two saccharides affected bile resistance in the mutants (Table 2). In addition, the *hldE*, *hldD*, and *waaC* mutants of NCTC 11168 and 81-176, which lacked all LOS sugars except Kdo, had greatly increased susceptibilities to bile acids. The susceptibility of the *waaF* mutant derived from 81-176 to bile acids was comparable to that of mutants that lacked all sugars except Kdo, although LOS of this strain contained a heptose and P/*P*Etn in addition to Kdo [25]. In contrast, the *waaF* mutant derived from NCTC 11168, which contained an additional glucose linked to a heptose, was more resistant to bile acids than the mutants derived from 81-176 (Table 2 and Fig. 2). The differences in the susceptibilities to bile acids of the *waaF* mutants derived from 81-176 and NCTC 11168 were reflected in their growth in chicken intestinal extracts (Fig. 3). In addition to a heptose linked to a Kdo in LOS of *C. jejuni*, one or more sugars might be essential for bile acid tolerance.

The migration of LOS produced by the isogenic *hldE* and *hldD* mutants on SDS-PAGE was slightly slower than that of LOS produced by the *waaC* mutant derived from NCTC 11168 and 81-176 (Fig. 2A). The *hldE* and *hldD* mutants were expected to lack the ability to generate ADP-l-*glycero*-d-*manno*-heptose. In the *rfaD* (a homolog of *hldD*) mutants of *E. coli* and *H. influenzae*, d-*glycero*-d-*manno*-heptose is incorporated into the inner core instead of l-*glycero*-d-*manno*-heptose, although the sugar chains are not extended further [32], [33]. GC-MS analysis showed that LOS produced by the isogenic *hldE* and *hldD* mutants contained Kdo residues, whereas other possible inner core components, including l-*glycero*-d-*manno*-heptose and d-*glycero*-d-*manno*-heptose, were not detected in this study. MALDI-TOF MS analysis in the negative ion linear mode showed that three residues were incorporated into LOS (Table S2), and the mass differences between the observed ions at approximately Δ*m*/*z* 220 suggested that the residues were Kdo. The results of SDS-PAGE and GC-MS analysis suggested that there were some modifications to the Kdo_2_-lipid A structure in the isogenic *hldE* and *hldD* mutants, such as a third Kdo addition, although future detailed analyses are necessary to determine the LOS OS structures of these mutants.

CPS is detectable on the surface of *C. jejuni* and is known to play an important role in serum resistance, epithelial cell invasion, and the onset of diarrheal disease [34]. In this study, CPS was detected in all LOS-truncated mutants, and bile resistance of CPS mutants was comparable to that of wild-type strains. These results suggest that CPS is not essential for bile resistance in *C. jejuni*, which was previously suggested by Jeon et al. [23].

Bile acids are surface active detergents with potent antimicrobial activities [15]. The binding of bile acids to membrane lipids is correlated to their hydrophobicities. Increased hydrophobicity may facilitate the accessibility of bile acids to membrane lipids, thus leading to membrane damage. The increased susceptibilities to bile acids due to severe truncations of LOS OS may have been observed because of the higher hydrophobicities conferred by the shorter LOS chains, which made the microbe more susceptible to bile acids (Table 2 and Fig. 5).

The bile acid concentration in chicken intestines varies at different detection sites. The bile acid concentration in jejunal contents is >10 mM, which is lethal to many non-enteric bacteria [35]. The concentration in the upper gastrointestinal tract is higher than that in the lower part. Lin et al. reported that the bile acid concentration in jejunal and cecal extracts were 14.0 mM and 0.17 mM, respectively [19]. This may explain why *C. jejuni* mainly colonizes the lower intestines of chickens [36]. In this study, the bile acid concentrations of the jejunal and ileal extracts were 8.5 mM and 4.9 mM, respectively. These bile acid concentrations did not affect the growth of NCTC 11168 and 81-176 in the extracts, although these strains were poor colonizers of chicken intestines in our laboratory. Thus, these experiments showed that jejunal and ileal extracts did not accurately replicate the intestinal environments of chickens. However, it has been shown that these systems can be used to evaluate the tolerance of *C. jejuni* strains to bile acids by simulating the conditions in intestinal tracts of chickens.

Inactivation of *hldE*, *hldD*, and *waaC* greatly increased the susceptibility to jejunal and ileal extracts from chickens in this study (Fig. 3). The *hldD* mutant derived from 11-164 can be killed or damaged by high concentration of bile acids in the jejunum and ileum of chickens, while colonization of the lower intestine by this mutant was lower than that by the wild-type strain. Overall, this study demonstrated that the addition of more than one sugar to Kdo facilitated bile acid tolerance and promoted the colonization of chicken intestinal tracts. These data should be useful for developing novel control measures to prevent *C. jejuni* colonization of chicken intestines. Inhibitors of the *C. jejuni* enzymes, bifunctional d-*glycero*-d-*manno*-heptose 7-phosphate kinase/d-*glycero*-d-*manno*-heptose 1-phosphate adenylyltransferase (HldE), ADP-d-*glycero*-d-*manno*-heptose epimerase (HldD), and heptosyltransferase I (WaaC), might act as potent anti-*Campylobacter* agents.

## Materials and Methods

### Bacterial strains, plasmids, and culture conditions

The bacterial strains and plasmids used in this study and their sources are listed in Table 1. MH broth and MH agar were used to grow the *C. jejuni* strains at 42°C under microaerophilic conditions, which were generated using AnaeroPack (Mitsubishi Gas Chemical Company, Inc., Tokyo, Japan) in an enclosed jar. *E. coli* DH5α was grown in Luria–Bertani broth or on LB agar (Becton Dickinson and Company) with or without ampicillin (100 µg/ml) and kanamycin (30 µg/ml) at 37°C.

### PCR

The PCR primers used in this study are shown in Table S4. PCR was performed in a volume of 50 µl containing 200 µM of each of the four deoxynucleoside triphosphates, 2 or 1 mM of MgCl_2_, 250 nM of primers, 50 ng of template DNA, and 1.25 U of Ex Taq DNA polymerase (Takara Bio Inc., Otsu, Japan) or Pyrobest DNA polymerase (Takara Bio). Amplifications were performed using iCycler (Bio-Rad Laboratories, Hercules, CA, USA). The cycling conditions varied depending on the estimated annealing temperatures of the primers and the expected size of the products. The size of the PCR products was confirmed by agarose gel electrophoresis.

### Construction of LOS mutants of *C. jejuni*

Isogenic mutants were constructed using a nonpolar chloramphenicol cassette [37]. The upstream and downstream regions of each target gene and the *cat* gene encoding chloramphenicol resistance from pUOA18 [38] were amplified independently and used as templates for overlapping extension PCR to join the three fragments [39]. The fragments produced were cloned into the pGEM-T Easy vector (Promega Corporation, Madison, WI, USA) to generate suicide vectors. Each suicide vector was introduced into NCTC 11168 or 81-176 by electroporation using a MicroPulser electroporator (Bio-Rad Laboratories), according to the manufacturer's instructions. Transformants were selected on MH agar containing 6 µg/ml chloramphenicol.

The construction of complemented strains of LOS-truncated mutants was based on a previous study [40]. Each target gene encoding the biosynthetic enzyme and promoter region of *cmeABC* was amplified independently and used as a template for overlapping extension PCR. The fragments produced were digested with EcoRI and BamHI and cloned into pUOA18Km. Each vector was introduced into the corresponding LOS-truncated mutant via triparental mating using Dh5α harboring the helper plasmid pRK2013 [41]. The complemented strain 164hldDc was selected using MH agar plates containing kanamycin (30 µg/ml) and chloramphenicol (6 µg/ml).

The genomic DNA from *C. jejuni* 168hldE, 168hldD, 168waaC, and 168waaF were used to transform *C. jejuni* 11-164 in order to generate the 164hldE, 164hldD, 164waaC, and 164waaF as described previously [42]. *hldD* was cloned into pUOA18Km and transformed into 164hldD for *trans*-complementation.

### Isolation of LOS and the OS fraction

LOS was obtained from each strain by hot phenol extraction followed by RNase, DNase, and protease treatments, as reported previously [43]. LOS samples were separated by tricine-SDS-PAGE [44] using a 16% (w/v) polyacrylamide separating gel and visualized by silver staining [45].

LOS samples were prepared for MALDI-TOF MS analyses using the following procedure. Each LOS band was excised from the separating gel of tricine-SDS-PAGE, ground, and homogenized using a spatula. Five volumes of distilled water was added to one volume of homogenate, and then incubated overnight at room temperature with shaking. After centrifugation at 5,000×*g* for 10 min, LOS was filtered from the supernatant through a 0.45-µm membrane filter (Toyo Roshi Kaisha, Ltd., Tokyo, Japan) and precipitated by performing two sets of centrifugation at 284,000×*g* for 8 h.

OS was released from LOS samples by mild acid hydrolysis in 1.5% acetic acid at 100°C for 2 h [46] and lyophilization. Lipid A was removed with CHCl_3_/MeOH/H_2_O (12∶8∶1) [47].

### LOS analysis by mass spectrometry

The structures of LOS and OS were analyzed by MALDI-TOF MS in the negative ion mode using a 4800 *plus* MALDI TOF/TOF analyzer (AB Sciex, Framingham, MA, USA). The ion-accelerating voltage was set at 20 kV. Freeze-dried LOS or OS was dissolved in 30% methanol and desalted with a few grains of a cation exchange resin, i.e., Dowex 50W-X2 (H^+^ form) (Wako Pure Chemical Industries, Ltd., Osaka, Japan). The solution (0.5 µl) was deposited on the target and covered with the same amount of the matrix solution containing 20 mg/ml of 2,5-dihydroxybenzoic acid (DHB, Bruker Daltonics Inc., Billerica, MA, USA) in 30% methanol with 0.1 M citric acid [48]. The mass spectrometer was tuned and calibrated using commercially available standard peptides in the reflector mode and proteins in the linear mode (Bruker Daltonics; Peptide calibration standard I containing angiotensin II, angiotensin I, substance P, bombesin, ACTH clip 1–17, ACTH clip 18–39, somatostatin 28; and Protein calibration standard I containing insulin, ubiquitin, cytochrome C, and myoglobin).

Sugar compositions of LOS and OS were determined as their alditol acetates by GC-MS [46].

### Susceptibility tests

MICs of the antimicrobials shown in Table 2 were determined by the standard microtiter broth dilution method using MH broth containing an inoculum of 10^6^ CFU/well, as described previously [49]. Bacterial growth was assessed after incubating the microtiter plates for 48 h at 42°C under microaerobic conditions. The MIC experiments were repeated three times using each strain tested in this study and each of the different antimicrobials, and the results of one representative experiment are shown.

### Growth assay using chicken intestinal extracts

Chicken intestinal extracts were prepared as described by Lin et al. [19]. In brief, jejunal and ileal contents of six 28-day-old chickens were obtained and pooled. Each sample was mixed with the same volume of MH broth and centrifuged at 10,000×*g* at 4°C for 30 min. The supernatant was filtered through a 0.45-µm membrane filter (Toyo Roshi Kaisha). To sequestrate bile acids, each chicken intestinal extract was pretreated with 5% (w/v) cholestyramine resin (Sigma-Aldrich Co., St. Louis, MO, USA) at room temperature for 1 h with intermittent vortexing [50]. After incubation, cholestyramine was removed by centrifugation and filtration. Filtration was performed using a 0.2-µm membrane filter (Toyo Roshi Kaisha). Total bile acid concentrations in each intestinal extract and cholestyramine-treated extracts were measured using the colorimetric Total Bile Acid Test Kit (Diazyme Laboratories, San Diego, CA, USA), according to the manufacturer's instruction. Bile acid concentrations were recorded as the arithmetic means of three independent analyses.

The growth of *C. jejuni* strains in the chicken intestinal extracts and cholestyramine-treated extracts was measured using the following procedure. Thirty microliters of the *C. jejuni* strain (approximately 5×10^8^ CFU/ml) and 300 µl of a 1∶10 dilution of each extract were mixed in a 96-well plate (in triplicate) and incubated at 42°C under microaerophilic conditions for 24 h. During incubation, 50 µl of the mixture was removed from each well at different time points (0, 6, 12, and 24 h after inoculation), serially diluted, and plated onto MH agar to enumerate the *Campylobacter* colonies in each sample. With each strain tested in this study, the growth assay was repeated three times with each of the chicken intestinal extract and the results of one representative experiment are shown.

### Chicken colonization experiments

To analyze the effect of *hldD* mutation on intestinal colonization by *C. jejuni*, *hldD* mutant and complementary strain were constructed using *C. jejuni* 11-164. The resistant strain 11-164 was selected using MH agar plates containing 100 µg/ml nalidixic acid and used as the wild-type strain to construct the mutant and complementary strains. The nalidixic acid-resistant phenotype of this strain facilitated the enumeration of viable *C. jejuni* cells in the cecal contents of experimentally infected chicks.

Newly hatched 1-day-old chicks were obtained from Nisseiken Co. Ltd. (Ome, Japan). Before use, these chicks were screened for *Campylobacter* by culturing cloacal swabs on MH agar plates containing *Campylobacter*-specific growth supplements (SR232E and SR117E; Oxoid Ltd., Basingstoke, UK). All chicks tested negative for *Campylobacter*. To compare colonization of strains, 54 three-day-old chicks were assigned to three groups (18 chicks/group). Each group was challenged with 10^6^ CFU of 11-164 wild type, 164hldD, or 164hldDc. Three chicks from each group were sacrificed at 1, 3, 5, 8, 10, and 12 days after inoculation, and their cecal contents were collected, serially diluted, and spread on MH agar plates supplemented with nalidixic acid. Plates were incubated at 42°C under microaerophilic conditions for 48 h, and *Campylobacter* colonies formed on each plate were counted.

These experiments were carried out in strict accordance with the guidelines of animal experimentation defined by the National Institute of Animal Health (NIAH) of Japan. The protocol was approved by the committee on the Ethics of Animal Experiments of the NIAH (Permit Number 10-027).

### Hydrophobicity test

Bacterial adherence to hydrocarbons was tested as described by Rosenberg et al. [51]. In brief, freshly grown *C. jejuni* cells on MH agar plates were harvested and washed twice using phosphate-buffered saline (PBS), centrifuged, and resuspended in the same buffer to produce an optical density (OD) of 0.5 at 600 nm. One milliliter of the suspension and 1 ml of n-octane (Wako Pure Chemical Industries) were placed in a borosilicate glass tube and mixed using a vortex mixer for 120 s. After 15 min of standing, the aqueous phase was transferred to a cuvette and OD_600_ was measured. The surface hydrophobicity (%) of the bacteria was calculated as follows: (1−final OD_600_ of the aqueous phase/initial OD_600_ of the cell suspension)×100. All tests were performed in triplicate.

### Statistical analysis

Differences in the results were tested using the two-tailed unpaired Student's *t* test. *P*<0.05 was considered statistically significant. The results of chicken colonization experiments and hydrophobicity tests are expressed as the means with error bars denoting the standard deviations of the means.

## Supporting Information

Figure S1 **CPS profiles of the** ***C. jejuni*** **wild-type strains (NCTC 11168 and 81-176) and mutants.** CPS samples were obtained from each strain by hot phenol extraction followed by RNase, DNase, and protease treatments. CPS samples were separated on a 4–20% Tris-glycine SDS-PAGE gel and visualized by Alcian blue staining.
(TIF)

Figure S2 **LOS profiles, motility, and cell surface hydrophobicity of the wild-type 11-164 and mutants.** (A) LOS samples were analyzed by tricine-SDS-PAGE using 16% tricine gels and visualized by silver staining. The positions of the protein size markers are indicated on the left of each panel. (B) Zones of motility of wild-type 11-164 and mutants in 0.4% soft MH agar. (C) Effect of LOS truncations on the cell surface hydrophobicity of the wild-type 11-164 and mutants. The percentage hydrophobicity of the bacterial surface was calculated as follows: (1−final OD_600_ of the aqueous phase/initial OD_600_ of the cell suspension)×100. Three independent experiments were performed using the same strains and conditions. The standard deviations are indicated by error bars.
(TIF)

Figure S3 **Growth properties in MH broth and survival in MH broth at pH 4.0 of the wild-type 11-164, 164hldD, and 164hldDc.** (A) To compare the growth kinetics of the mutants of 11-164 with that of the wild-type strain, the cultures were inoculated separately into MH broth at an initial cell density of 5×10 CFU/ml. The cultures were incubated at 42°C under microaerobic conditions. Aliquots of the cultures were collected at different time points (0, 8, 24, 32, 48, and 72 h), serially diluted, and plated onto MH agar plates to enumerate the bacterial colonies. Three independent experiments were performed using the same strains and conditions. (B) Bacterial cultures were inoculated separately into MH broth (pH 4.0) at an initial cell density of 1×10^8^ CFU/ml and incubated at 42°C under microaerobic conditions. Aliquots of the cultures were collected at different time points (0, 0.5, 1, and 2 h), serially diluted, and plated onto MH agar plates to enumerate the bacterial colonies. Three independent experiments were performed using the same strains and conditions.
(TIF)

Table S1 **Major ions in the negative reflectron mode MALDI-TOF mass spectra and the proposed compositions of the OS chains of 168cj1135, 168cj1136, and168cj1138.**
(DOCX)

Table S2 **Major ions in the negative linear mode MALDI-TOF mass spectra and the proposed compositions of the intact LOS chains of 168hldE, 168hldD, 817hldE, 817hldD, and 164hldD.**
(DOCX)

Table S3 **Antimicrobial susceptibility of wild-type 11-164 and mutants.**
(DOCX)

Table S4 **Primers used in this study.**
(DOCX)
