# Supplementary figures and images for: Effects of Lipooligosaccharide Inner Core Truncation on Bile Resistance and Chick Colonization by Campylobacter jejuni
Source: PLoS One. 2013 Feb 20;8(2):e56900. doi: 10.1371/journal.pone.0056900 (PMC3577681; doi:10.1371/journal.pone.0056900)

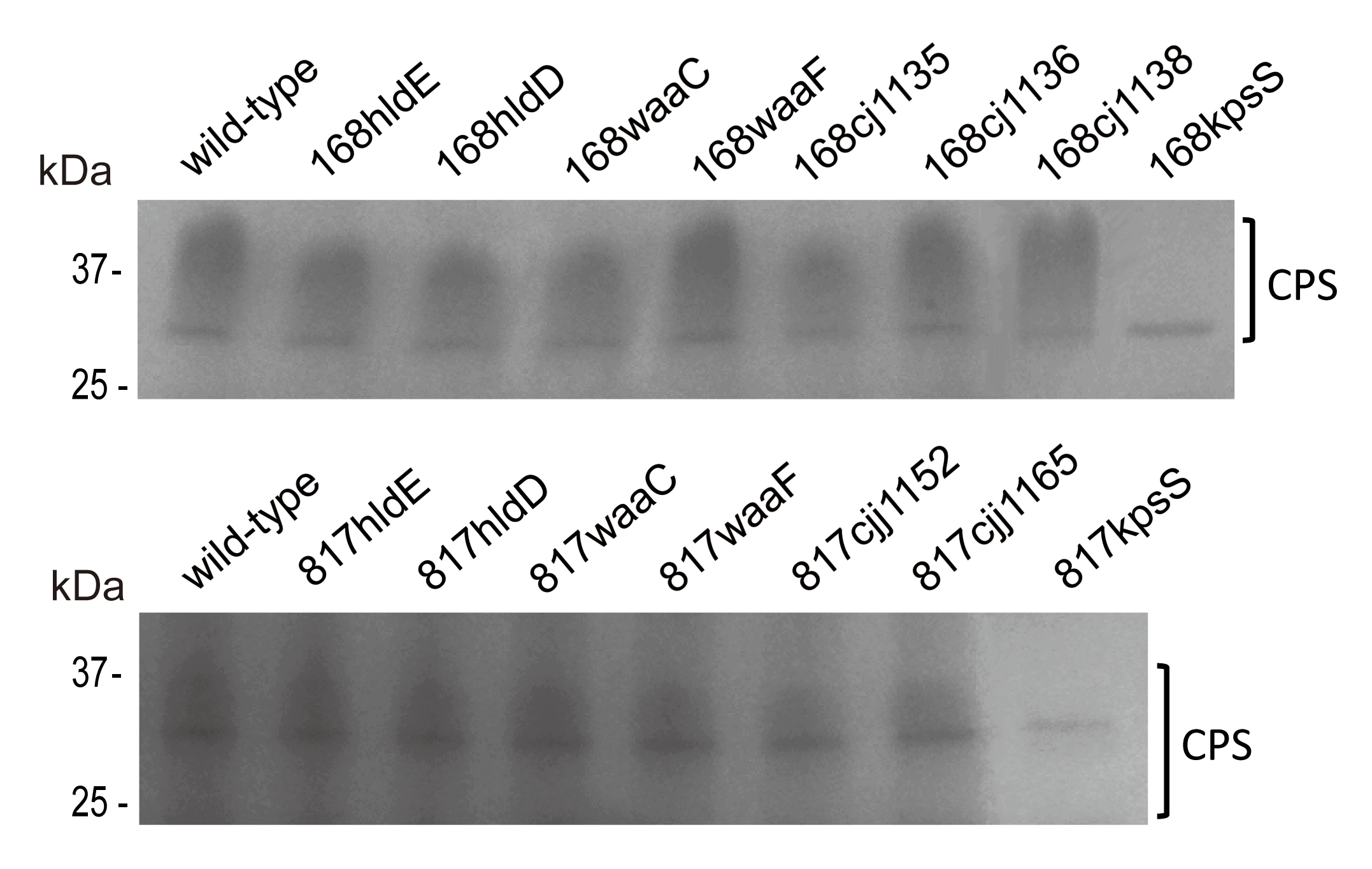

Supplement: Figure S1 — CPS profiles of the C. jejuni wild-type strains (NCTC 11168 and 81-176) and mutants. CPS samples were obtained from each strain by hot phenol extraction followed by RNase, DNase, and protease treatments. CPS samples were separated on a 4–20% Tris-glycine SDS-PAGE gel and visualized by Alcian blue staining. (TIF) [file pone.0056900.s001.tif]

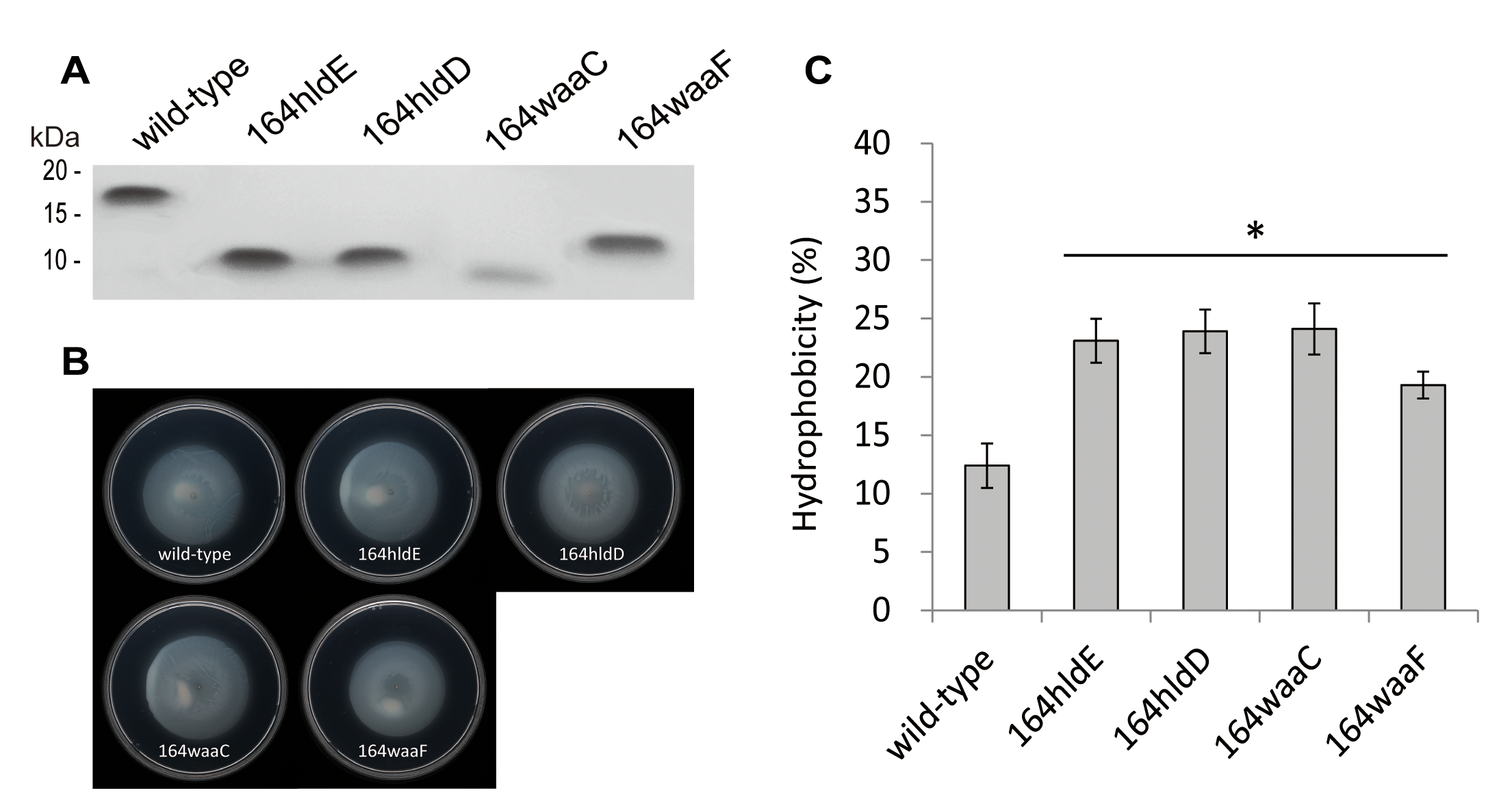

Supplement: Figure S2 — LOS profiles, motility, and cell surface hydrophobicity of the wild-type 11-164 and mutants. (A) LOS samples were analyzed by tricine-SDS-PAGE using 16% tricine gels and visualized by silver staining. The positions of the protein size markers are indicated on the left of each panel. (B) Zones of motility of wild-type 11-164 and mutants in 0.4% soft MH agar. (C) Effect of LOS truncations on the cell surface hydrophobicity of the wild-type 11-164 and mutants. The percentage hydrophobicity of the bacterial surface was calculated as follows: (1−final OD600 of the aqueous phase/initial OD600 of the cell suspension)×100. Three independent experiments were performed using the same strains and conditions. The standard deviations are indicated by error bars. (TIF) [file pone.0056900.s002.tif]

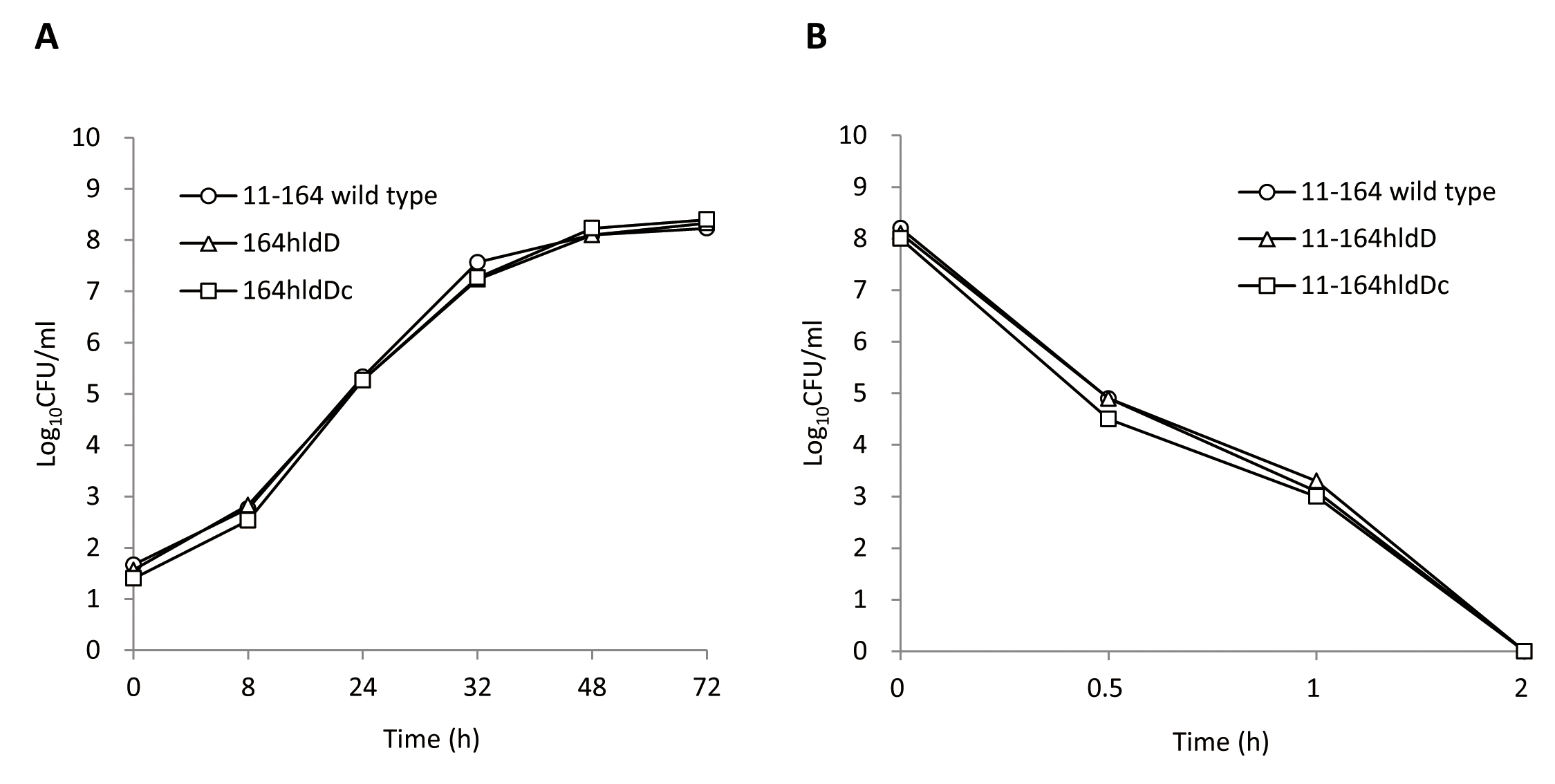

Supplement: Figure S3 — Growth properties in MH broth and survival in MH broth at pH 4.0 of the wild-type 11-164, 164hldD, and 164hldDc. (A) To compare the growth kinetics of the mutants of 11-164 with that of the wild-type strain, the cultures were inoculated separately into MH broth at an initial cell density of 5×10 CFU/ml. The cultures were incubated at 42°C under microaerobic conditions. Aliquots of the cultures were collected at different time points (0, 8, 24, 32, 48, and 72 h), serially diluted, and plated onto MH agar plates to enumerate the bacterial colonies. Three independent experiments were performed using the same strains and conditions. (B) Bacterial cultures were inoculated separately into MH broth (pH 4.0) at an initial cell density of 1×10^8^ CFU/ml and incubated at 42°C under microaerobic conditions. Aliquots of the cultures were collected at different time points (0, 0.5, 1, and 2 h), serially diluted, and plated onto MH agar plates to enumerate the bacterial colonies. Three independent experiments were performed using the same strains and conditions. (TIF) [file pone.0056900.s003.tif]
