# Supplementary material for: Effects of Lipooligosaccharide Inner Core Truncation on Bile Resistance and Chick Colonization by Campylobacter jejuni
Source: PLoS One. 2013 Feb 20;8(2):e56900. doi: 10.1371/journal.pone.0056900 (PMC3577681; doi:10.1371/journal.pone.0056900)
Supplement: Table S1 — Major ions in the negative reflectron mode MALDI-TOF mass spectra and the proposed compositions of the OS chains of 168cj1135, 168cj1136, and168cj1138. (DOCX) [file pone.0056900.s004.docx]

| Table S1. Major ions in the negative reflectron mode MALDI-TOF mass spectra and the | | | |
| --- | --- | --- | --- |
| proposed compositions of the OS chains of 168cj1135, 168cj1136, and168cj1138 | | | |
| Strain | Proposed composition | *m/z* of 〔M-H〕^－^ | |
|  |  | Observed | Calculated |
| 168cj1135 | Hep_2_・PEtn_1_・Kdo_2_ | 744.3 | 744.2 |
|  | Hep_2_・P_1_・Kdo_2_ | 701.2 | 701.1 |
| 168cj1136 | Glc_2_・Hep_2_・PEtn_1_・Kdo_2_ | 1068.3 | 1068.3 |
|  | Glc_2_・Hep_2_・P_1_・Kdo_2_ | 1025.2 | 1025.2 |
| 168cj1138 | Gal_1_・Glc_2_・Hep_2_・PEtn_1_・Kdo_2_ | 1230.5 | 1230.4 |
|  | Gal_1_・Glc_2_・Hep_2_・P_1_・Kdo_2_ | 1187.4 | 1187.3 |
