# Supplementary material for: Effects of Lipooligosaccharide Inner Core Truncation on Bile Resistance and Chick Colonization by Campylobacter jejuni
Source: PLoS One. 2013 Feb 20;8(2):e56900. doi: 10.1371/journal.pone.0056900 (PMC3577681; doi:10.1371/journal.pone.0056900)
Supplement: Table S2 — Major ions in the negative linear mode MALDI-TOF mass spectra and the proposed compositions of the intact LOS chains of 168hldE, 168hldD, 817hldE, 817hldD, and 164hldD. (DOCX) [file pone.0056900.s005.docx]

| Table S2. Major ions in the negative linear mode MALDI-TOF mass spectra and the proposed | | | |
| --- | --- | --- | --- |
| compositions of the intact LOS chains of 168hldE, 168hldD, 817hldE, 817hldD,and 164hldD | | | |
| Strain | Proposed composition | *m/z* of 〔M-H-H_2_O〕^－^ | |
|  |  | Observed | Calculated |
| 168hldE | Kdo_3_・lipid A | 2566 | 2565 |
|  | Kdo_2_・lipid A | 2344 | 2345 |
|  | Kdo_1_・lipid A | 2125 | 2124 |
| 168hldD | Kdo_3_・lipid A | 2565 | 2565 |
|  | Kdo_2_・lipid A | 2345 | 2345 |
|  | Kdo_1_・lipid A | 2125 | 2124 |
| 817hldE | Kdo_3_・lipid A | 2565 | 2565 |
|  | Kdo_2_・lipid A | 2345 | 2345 |
|  | Kdo_1_・lipid A | 2125 | 2124 |
| 817hldD | Kdo_3_・lipid A | 2566 | 2565 |
|  | Kdo_2_・lipid A | 2346 | 2345 |
|  | Kdo_1_・lipid A | 2125 | 2124 |
| 164hldD | Kdo_3_・lipid A | 2566 | 2565 |
|  | Kdo_2_・lipid A | 2346 | 2345 |
|  | Kdo_1_・lipid A | 2125 | 2124 |
