# Supplementary material for: Effects of Lipooligosaccharide Inner Core Truncation on Bile Resistance and Chick Colonization by Campylobacter jejuni
Source: PLoS One. 2013 Feb 20;8(2):e56900. doi: 10.1371/journal.pone.0056900 (PMC3577681; doi:10.1371/journal.pone.0056900)
Supplement: Table S3 — Antimicrobial susceptibility of wild-type 11-164 and mutants. (DOCX) [file pone.0056900.s006.docx]

| Table S3. Antimicrobial susceptibility of wild-type 11-164 and mutants | | | | | | | | | | |
| --- | --- | --- | --- | --- | --- | --- | --- | --- | --- | --- |
| Antimicrobial^a^ | MIC (μg/ml) | | | | | | | | | |
|  | wild-type | | 164hldE | | 164hldD | | 164waaC | | 164waaF | |
| **Ox gall** | 50,000 |  | 12,500 |  | 12,500 |  | 12,500 |  | 25,000 |  |
| **Ox bile extract** | 25,000 |  | 6,250 |  | 6,250 |  | 6,250 |  | 12,500 |  |
| **Cholic acid** | 6,250 |  | 1,560 |  | 1,560 |  | 1,560 |  | 3,130 |  |
| **Deoxycholic acid** | >10,000 |  | 625 |  | 625 |  | 625 |  | 1,250 |  |
| **Taurocholic acid** | >100,000 |  | 12,500 |  | 12,500 |  | 12,500 |  | 50,000 |  |
| **SDS** | 100 |  | 50 |  | 50 |  | 50 |  | 50 |  |
| Ampicillin | >50 |  | >50 |  | >50 |  | >50 |  | >50 |  |
| Cefsulodin | 25 |  | 25 |  | 25 |  | 25 |  | 25 |  |
| Gentamicin | 0.313 |  | 0.313 |  | 0.313 |  | 0.313 |  | 0.313 |  |
| Tetracycline | 12.5 |  | 12.5 |  | 12.5 |  | 12.5 |  | 12.5 |  |
| **Erythromycin** | 0.5 |  | 0.0625 |  | 0.0625 |  | 0.0625 |  | 0.0625 |  |
| Trimethoprim | 250 |  | 250 |  | 250 |  | 250 |  | 250 |  |
| Nalidixic acid | >10 |  | >10 |  | >10 |  | >10 |  | >10 |  |
| Enrofloxacin | 0..0625 |  | 0..0625 |  | 0..0625 |  | 0..0625 |  | 0..0625 |  |
| **Rifampin** | 200 |  | 200 |  | 100 |  | 100 |  | 100 |  |
| **Polymyxin B** | 10 |  | 5 |  | 5 |  | 5 |  | 5 |  |
| ^a^Bold-faced types indicate that the differences in MICs observed between 11-164 wild-type strain and any mutants. | | | | | | | | | | |
