# Supplementary material for: Effects of Lipooligosaccharide Inner Core Truncation on Bile Resistance and Chick Colonization by Campylobacter jejuni
Source: PLoS One. 2013 Feb 20;8(2):e56900. doi: 10.1371/journal.pone.0056900 (PMC3577681; doi:10.1371/journal.pone.0056900)
Supplement: Table S4 — Primers used in this study. (DOCX) [file pone.0056900.s007.docx]

| Table S4. Primers used in this study | |  |  |
| --- | --- | --- | --- |
| Primer name | Sequence (5′→3′)^a^ | Target | Origin^b^ |
| CHEF | TGCTCGGCGGTGTTCCTTT | *cat* | pUOA18 |
| CHER | GCGCCCTTTAGTTCCTAAAG |  | pUOA18 |
| GMHA.1 | CAGGAAAAGCCGAACAAGAA | upstream region of *gmhA* | NCTC 11168 |
| 81GMHA.1 | TCTGCCCAGCACCCAAG |  | 81-176 |
| GMHA.2 | AAAGGAACACCGCCGAGCATGTGCCAAGCGATGATACAG |  | NCTC 11168 |
| 81GMHA.2 | AAAGGAACACCGCCGAGCATGTCGTGCCAAGTGATGATAC |  | 81-176 |
| GMHA.3 | CTTTAGGAACTAAAGGGCGCACCGTTTCCGCAAATTAAAA | downstream region of *gmhA* | NCTC 11168 |
| 81GMHA.3 | CTTTAGGAACTAAAGGGCGCTTTTAAAATTTCACTGGCTTGAGC |  | 81-176 |
| GMHA.4 | AGGCGAGAGTCGTCCTGTAA |  | NCTC 11168 and 81-176 |
| HLDE.1 | TGCTAAAGCCTTGCGTTCTT | upstream region of *hldE* | NCTC 11168 and 81-176 |
| HLDE.2 | AAAGGAACACCGCCGAGCAGGTGCAGATATCGTTTCAAGG |  | NCTC 11168 |
| 81HLDE.2 | AAAGGAACACCGCCGAGCATTTCAAAGGTTGAATTAATCGAC |  | 81-176 |
| HLDE.3 | CTTTAGGAACTAAAGGGCGCATACGCGAACAATCACACCA | downstream region of *hldE* | NCTC 11168 and 81-176 |
| HLDE.4 | ATCGCCCTTGATTCAAAATG |  | NCTC 11168 and 81-176 |
| HLDD.1 | TCACTCCCCTTAGGATCAACC | upstream region of *hldD* | NCTC 11168 and 81-176 |
| HLDD.2 | AAAGGAACACCGCCGAGCATTTTTGAAAAGGAAGTAAATGCTTG |  | NCTC 11168 and 81-176 |
| HLDD.3 | CTTTAGGAACTAAAGGGCGCCAAATTTAAAGCTAGTTGCGAACC | downstream region of *hldD* | NCTC 11168 and 81-176 |
| HLDD.4 | GATTTCGAATTTTGTGATGGAA |  | NCTC 11168 and 81-176 |
| WAAC.1 | TTCTTTTTGCCACTGAAGTGC | upstream region of *waaC* | NCTC 11168 and 81-176 |
| WAAC.2 | AAAGGAACACCGCCGAGCATAAATTGCAAAACCACGGCAC |  | NCTC 11168 and 81-176 |
| WAAC.3 | CTTTAGGAACTAAAGGGCGCACCGCAATGCTTTTCAAACT | downstream region of *waaC* | NCTC 11168 and 81-176 |
| WAAC.4 | TGATGATTTTGCGGTGTTTT |  | NCTC 11168 and 81-176 |
| WAAF.1 | ACTTGCAAAAGTGCCCAAAT | upstream region of *waaF* | NCTC 11168 |
| 81WAAF.1 | AATTTCTACTTGCAAAAGTGCC |  | 81-176 |
| WAAF.2 | AAAGGAACACCGCCGAGCAGCCATTACCGTATCGCCTAA |  | NCTC 11168 |
| 81WAAF.2 | AAAGGAACACCGCCGAGCAAGCAGGCGAAGCCATC |  | 81-176 |
| WAAF.3 | CTTTAGGAACTAAAGGGCGCCCTGCCCTTTAAAACACCAC | downstream region of *waaF* | NCTC 11168 and 81-176 |
| WAAF.4 | TGTCTAGGGCTTAGCGGAAA |  | NCTC 11168 and 81-176 |
| 1135.1 | AAGTAATGCTTTGATCATCCC | upstream region of *Cj1135* | NCTC 11168 |
| 1135.2 | AAAGGAACACCGCCGAGCATAAAGAATTTAAACATTCAAGCAAGG |  | NCTC 11168 |
| 1135.3 | CTTTAGGAACTAAAGGGCGCATGATGGAGTGTTTAAAAGGC | downstream region of *cj1135* | NCTC 11168 |
| 1135.4 | TCTATACCTACATTTCTAGCACTAC |  | NCTC 11168 |
| 1136.1 | TAGGGAAGATAGTGAGTTTG | upstream region of *cj1136* | NCTC 11168 |
| 1136.2 | AAAGGAACACCGCCGAGCATTTTCATCTGTACTACCATC |  | NCTC 11168 |
| 1136.3 | CTTTAGGAACTAAAGGGCGCTGATCTTTGGAAAATTTGGC | downstream region of *cj1136* | NCTC 11168 |
| 1136.4 | ACATGAGCGATATAGAATGC |  | NCTC 11168 |
| 1138.1 | ACTTTAGGGTGTTCTAGC | upstream region of *cj1138* | NCTC 11168 |
| 1138.2 | AAAGGAACACCGCCGAGCATGATTGATTACACTATCTAAAC |  | NCTC 11168 |
| 1138.3 | CTTTAGGAACTAAAGGGCGCAGATCCTTTGTGTATTAAAGAG | downstream region of *cj1138* | NCTC 11168 |
| 1138.4 | AACACAACCTTTGTATACCC |  | NCTC 11168 |
| CJJ1152.1 | TTGATCATCCCTGTTTATGC | upstream region of *cjj1152* | 81-176 |
| CJJ1152.2 | AAAGGAACACCGCCGAGCATCAAGCAAAGTTTGCTCAGC |  | 81-176 |
| CJJ1152.3 | CTTTAGGAACTAAAGGGCGCAAGATAGTGAATTTGTGGCTAG | downstream region of *cjj1152* | 81-176 |
| CJJ1152.4 | TTAATTTATACTCCCCACTAAAG |  | 81-176 |
| CJJ1165.1 | TATGCACATAAGTGCGG | upstream region of *cjj1165* | 81-176 |
| CJJ1165.2 | AAAGGAACACCGCCGAGCAAGACAACAGATATTAATTCCAC |  | 81-176 |
| CJJ1165.3 | CTTTAGGAACTAAAGGGCGCATTTTTAGCATCAATAGCCTTAAAG | downstream region of *cjj1165* | 81-176 |
| CJJ1165.4 | ACTTAATATGCTTTGTCTAGGG |  | 81-176 |
|  |  | *Continued on following page* | |
| Table S4. -*continued* | |  |  |
| Primer name | Sequence (5′→3′)^a^ | Target | Origin^b^ |
| PUOA18F | AATCCCAGTTTGTCGCACTG | pUOA18 plasmid | pUOA18 |
| PUOA18R | CATTTATCCTCCGTAAATTC |  | pUOA18 |
| KmF | CTAAAACAATTCATCCAGTAAAATATAATAT | *aphA-3* | pMW10 |
| KmR | ATGGCTAAAATGAGAATATCACC |  | pMW10 |
| HLDEF | GGAATTCCTTTATCATTTTTTATCCTTAATC | cds of *hldE* | NCTC 11168 |
| HLDER | TTAATTTTTCAAGGCAAAACCATGCTTGAGTTTTTAAGTCAGC |  | NCTC 11168 |
| HLDDF | GGAATTCCTGCTGACTTAAAAACTCAAGCA GGAATTCCTGCTGACTTAAAAACTCAAGCA | cds of *hldD* | NCTC 11168 |
| HLDDR | TTAATTTTTCAAGGCAAAACCATGAAAATAGCAATCACAGG CGGGATCCCGCTGATATTGGCACTTTGA |  | NCTC 11168 |
| WAACF | TTAATTTTTCAAGGCAAAACCATGAAAATAGCAATTGTTCGTTTG | cds of *waaC* | NCTC 11168 |
| WAACR | GGAATTCC TCACTATTTTTCATTAAGTAAGCC |  | NCTC 11168 |
| WAAFF | TTAATTTTTCAAGGCAAAACCATGAAAATTTTTATACATCTTCCCAC | cds of *waaF* | NCTC 11168 |
| WAAFR | GGAATTCC AGATCATAGATGAGAGTTTTTAAG |  | NCTC 11168 |
| 1135F | TTAATTTTTCAAGGCAAAACCATGAATCTAAAGCAAATAAGTG | cds of *cj1135* | NCTC 11168 |
| 1135R | GGAATTCCATGTTTTATCTCCAAGTTGTTTTTTC |  | NCTC 11168 |
| 1136F | TTAATTTTTCAAGGCAAAACCATGAAAAAAGTAGGTGTAGTAATC | cds of *cj1136* | NCTC 11168 |
| 1136R | GGAATTCCAAATCATTTTGCAAAGTTGATTTTATC |  | NCTC 11168 |
| 1138F | TTAATTTTTCAAGGCAAAACCATGAAAACCGTAGGTGTAGT | cds of *cj1138* | NCTC 11168 |
| 1138R | GGAATTCCATGGAAAAAATTTTTATATAATTTAATTCC |  | NCTC 11168 |
| CJJ1152F | TTAATTTTTCAAGGCAAAACCATGAATCTAAAGCAAATAAGCG | cds of *cjj1152* | 81-176 |
| CJJ1152R | GGAATTCC TATTTTCTTCATGTTTTATCTCCAAG |  | 81-176 |
| CJJ1165F | GGAATTCCAATTAATTAAAATTATACTTCACGTC | cds of *cjj1165* | 81-176 |
| CJJ1165R | TTAATTTTTCAAGGCAAAACCATGAAAGTTTTTATCATCAACTTAG |  | 81-176 |
| 164HLDDF | GGAATTCCTGCTGACTTAAAAACTCAAGCA | cds of *hldD* | NCTC 11168 |
| 164HLDDR | CGGGATCCCGCTGATATTGGCACTTTGA |  | NCTC 11168 |
| CMEF | CGGGATCCCGTGGAATCAATAGCTCCAAAGCTTAA | promoter region of CmeABC | 81-176 |
| CMER | GGTTTTGCCTTGAAAAATTAA |  | 81-176 |
| ^a^ Single and double underlined regions indicate the complementary sequences of CHEF or CFER primers, and CMER primer, respectively. | | | |
| ^b^The origins of genomic DNA or plasmids used as templates for PCR amplifications. | | | |
